# Supplementary material for: Growth Mode and Physiological State of Cells Prior to Biofilm Formation Affect Immune Evasion and Persistence of Staphylococcus aureus
Source: Microorganisms. 2020 Jan 12;8(1):106. doi: 10.3390/microorganisms8010106 (PMC7023439; doi:10.3390/microorganisms8010106)
Supplement: Supplementary file 1 [file microorganisms-08-00106-s001.zip › Supplementary Figures.docx]

**Supplementary material**

***
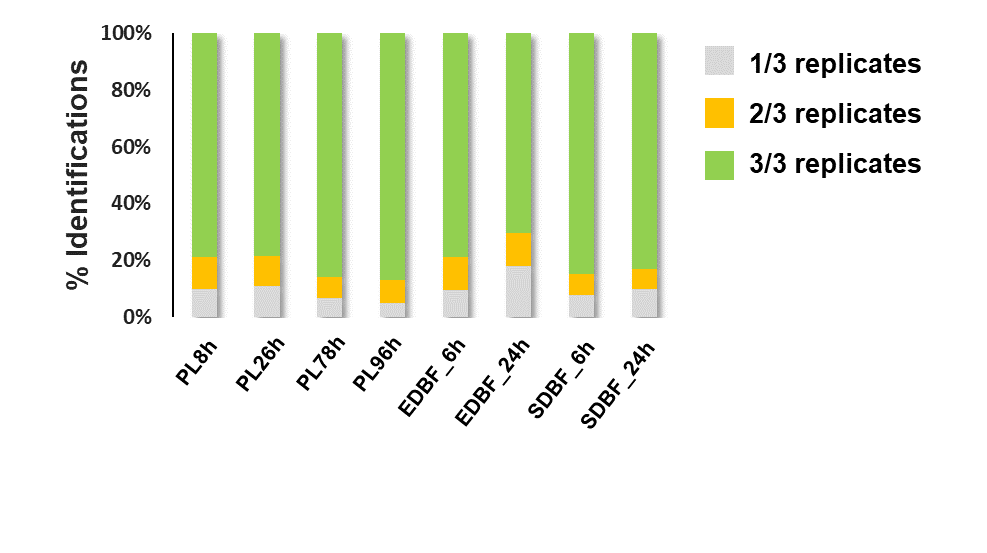
***

**Figure S1:** Distribution of the identified proteins in different replicate samples.

**
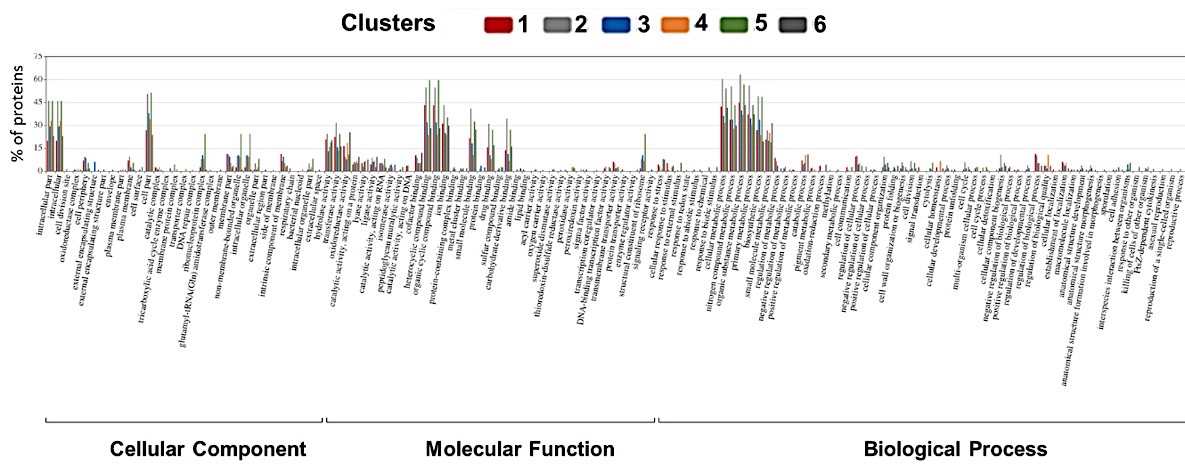
**

**Figure S2**: Distribution of the proteins associated with clusters 1 – 6 (heatmap shown in Figure 3) as a function of their GO terms.

**
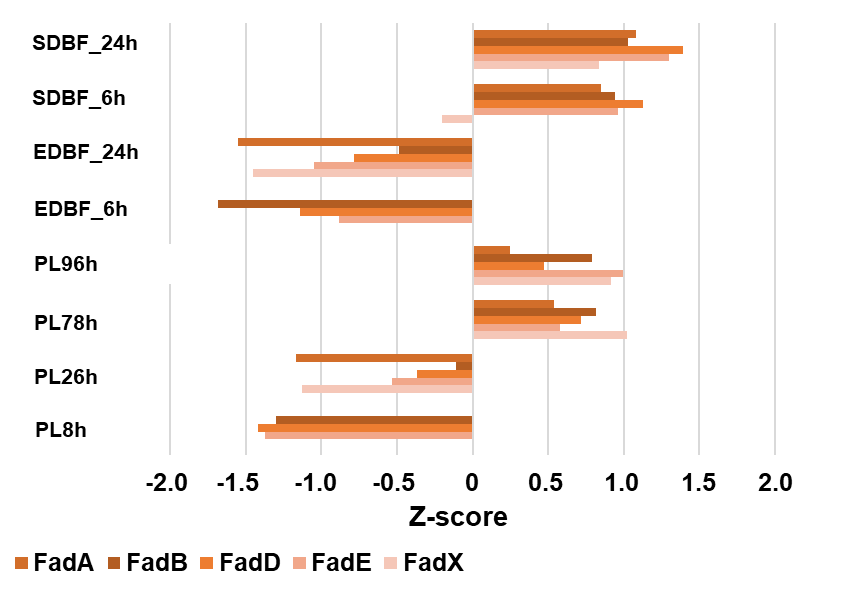

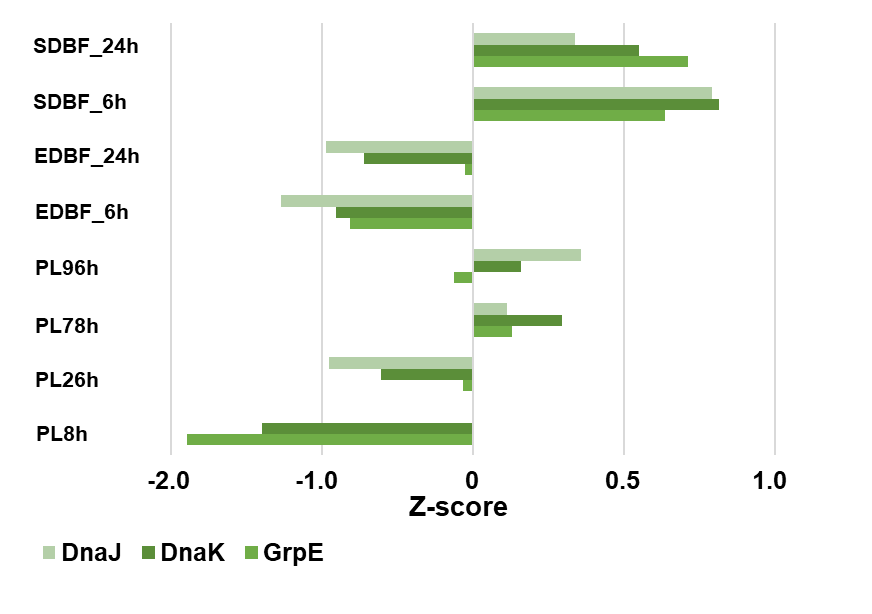
**

**Figure S3**: Abundances of the fadABDEX (A) and dnaJ-dnaK-grpE (B) operon-coded proteins in planktonic and biofilm cells at the indicated time points.
