# Supplementary material for: Growth Mode and Physiological State of Cells Prior to Biofilm Formation Affect Immune Evasion and Persistence of Staphylococcus aureus
Source: Microorganisms. 2020 Jan 12;8(1):106. doi: 10.3390/microorganisms8010106 (PMC7023439; doi:10.3390/microorganisms8010106)
Supplement: Supplementary file 1 [file microorganisms-08-00106-s001.zip › Table_S4.docx]

**Table S4.** Fold-changes of known virulence-, adhesion- and resistance-associated proteins on planktonic (PL) and biofilm (BF) cell surfaces at indicated time points. ED and SD, biofilm formation initiated with exponential (2 h) and stationary phase cells (72 h), respectively. Color scale, light red to red refers to increased protein abundance folds. Cells in grey, protein in question was below detection limit.


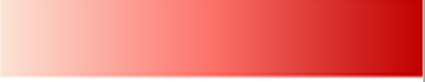


**0 → 75**

| **Acc. no.** | **Virulence factor / Adhesin** | **Fold-changes** | | | | | | | | | | | | | |
| --- | --- | --- | --- | --- | --- | --- | --- | --- | --- | --- | --- | --- | --- | --- | --- |
|  |  | **P=PL (h)** | | **E = EDBF and S=SDBF (h)** | | | | | | | **E = EDBF, S=SDBF, P=PL (h)** | | | | |
|  |  | **P8/ P96** | **P96/ P8** | **E6/ E24** | **S6/ S24** | **E24/ E6** | **S24/S6** | **E24/ S24** | **S24/ E24** | **E6/ P8** | **S6/ P78** | **E24/ P26** | **S24/ P96** | **P26/ E24** | **P96/S24** |
| WP_000594516.1 | HlgAB subunit A | 1.8 | 0.6 | 8.1 | 1.9 | 0.1 | 0.5 | 0.0 | 25.0 | 2.9 | 7.0 | 0.3 | 9.0 | 3.8 | 0.1 |
| WP_001056917.1 | HlgAB/HlgCB subunit B | P8 |  | E6 |  | 0.0 | 1.0 |  |  | P8 | 1.0 |  |  | 1.0 | 1.0 |
| WP_000595617.1 | Gamma-hemolysin subunit B | 60.6 | 0.0 | 4.6 | 0.7 | 0.2 | 1.4 | 4.9 | 0.2 | 15.5 | 0.2 | 0.2 | 0.7 | 4.4 | 1.4 |
| WP_000669728.1 | Protein MAP-domain containing | 19.7 | 0.1 | 3.0 | 1.4 | 0.3 | 0.7 | 1.7 | 0.6 | 7.8 | 0.7 | 0.1 | 1.5 | 10.2 | 0.7 |
| WP_000728713.1 | Immunoglobulin G-binding protein SpA | 8.3 | 0.1 | 2.9 | 0.5 | 0.3 | 1.8 | 0.5 | 1.9 | 5.8 | 0.8 | 0.4 | 3.7 | 2.4 | 0.3 |
| WP_000792567.1 | Immunoglobulin-binding protein Sbi | 74.8 | 0.0 | 11.5 | 1.1 | 0.1 | 0.9 | 5.2 | 0.2 | 60.2 | 0.5 | 0.9 | 1.0 | 1.1 | 1.0 |
| WP_000791398.1 | Leukocidin-like protein 2 | 21.8 | 0.1 | 4.5 | 1.1 | 0.2 | 0.9 | 4.0 | 0.3 | 11.0 | 0.3 | 0.4 | 0.6 | 2.6 | 1.6 |
| WP_000717395.1 | Secretory antigen SsaA2 | 12.4 | 0.1 | 5.0 | 0.9 | 0.2 | 1.1 | 6.2 | 0.2 | 12.4 | 0.3 | 0.9 | 0.4 | 1.2 | 2.5 |
| WP_000745926.1 | Clumping factor B ClfB | 30.5 | 0.0 | 5.5 | 0.7 | 0.2 | 1.5 | 1.5 | 0.7 | 57.4 | 1.9 | 1.5 | 6.8 | 0.7 | 0.2 |
| WP_077670278.1 | Clumping factor A ClfA | 20.0 | 0.1 | 2.5 | 0.4 | 0.4 | 2.3 | 0.4 | 2.3 | 8.6 | 1.4 | 0.6 | 7.8 | 1.7 | 0.1 |
| WP_000751265.1 | Immunodominant antigen IsaA | 32.3 | 0.0 | 7.5 | 1.9 | 0.1 | 0.5 | 1.0 | 1.0 | 34.3 | 9.7 | 1.0 | 4.4 | 1.0 | 0.2 |
| WP_001077096.1 | Immunodominant antigen IsaB | 0.5 | 1.9 | 4.7 | 1.4 | 0.2 | 0.7 | 0.0 | 58.5 | 1.1 | 37.5 | E24 | 13.2 | E24 | 0.1 |
| WP_000769723.1 | Peptidase propeptide YPEB/lipoprotein | 23.0 | 0.0 | 6.2 | 0.3 | 0.2 | 3.6 | 0.4 | 2.9 | 28.1 | 2.6 | 0.9 | 13.1 | 1.1 | 0.1 |
| WP_000825534.1 | Met ABC transporter NLPA/lipoprotein | 1.4 | 0.7 | 0.6 | 0.5 | 1.6 | 2.2 | 0.3 | 3.9 | 1.0 | 1.1 | 1.0 | 6.2 | 1.0 | 0.2 |
| WP_001033875.1 | 5'-nucleotidase, lipoprotein | 36.4 | 0.0 | 7.6 | 1.5 | 0.1 | 0.7 | 4.3 | 0.2 | 24.1 | 0.4 | 0.5 | 0.7 | 2.2 | 1.4 |
| WP_001074521.1 | Bifunctional autolysin Atl | 16.3 | 0.1 | 5.9 | 1.0 | 0.2 | 1.0 | 0.5 | 1.9 | 8.8 | 2.1 | 0.3 | 2.9 | 3.2 | 0.4 |
| WP_001170274.1 | Autolysin Sle1 | 6.2 | 0.2 | 2.7 | S6 | 0.4 | S6 |  |  | 1.8 | 0.5 | 0.4 | P96 | 2.3 | P96 |
| WP_029051775.1 | Matrix-binding protein Ebh |  | P96 | E6 |  |  |  |  |  | 1.5 | S6 | P26 | P96 | P26 | P96 |
| WP_038413132.1 | Fibronectin-binding protein FnbA | 20.0 | 0.1 | 3.7 | 1.4 | 0.3 | 0.7 | 2.4 | 0.4 | 15.3 |  | 2.2 | 1.7 | 0.5 | 0.6 |
| WP_038413163.1 | Collagen adhesin | 6.6 | 0.2 | 2.8 | 0.7 | 0.4 | 1.4 | 0.7 | 1.4 | 10.4 | 2.0 | 1.2 | 5.2 | 0.8 | 0.2 |
| WP_001151905.1 | Cell-wall-anchored protein SasF | 16.8 | 0.1 | 2.8 | 0.6 | 0.4 | 1.6 | 0.9 | 1.1 | 8.8 | 1.0 | 0.6 | 3.3 | 1.8 | 0.3 |
| WP_000934494.1 | MSCRAMM family adhesin SdrD | 25.8 | 0.0 | 10.1 | 1.7 | 0.1 | 0.6 | 2.2 | 0.5 | 40.9 | 18.0 | 2.3 | 1.8 | 0.4 | 0.5 |
| WP_077670283.1 | MSCRAMM family adhesin SdrE (part.) | 63.9 | 0.0 | 12.1 | 2.0 | 0.1 | 0.5 | 1.8 | 0.6 | 75.1 | 10.1 | 0.8 | 3.6 | 1.3 | 0.3 |
| WP_077670284.1 | MSCRAMM family adhesin - SdrC | P8 |  | 21.2 | 2.5 | 0.1 | 0.4 | 1.9 | 0.5 | 1.7 | E6 | 2.4 | S24 | 0.4 | S24 |
| WP_001041575.1 | Heme uptake protein IsdB | 4.3 | 0.2 | 1.8 | 2.4 | 0.6 | 0.4 | 0.8 | 1.3 | 2.1 | 5.5 | 0.9 | 1.5 | 1.1 | 0.7 |
| WP_000728052.1 | Matrix protein-binding adhesin Emp | P8 |  | 12.3 |  | 0.1 |  | E24 |  | 1.7 | P78 | 0.3 |  | 2.9 |  |
| WP_000782130.1 | Foldase protein PrsA | 3.9 | 0.3 | 1.00 | 1.6 | 1.0 | 0.6 | 1.6 | 0.64 | 1.1 | 2.4 | 1.1 | 2.69 | 1.0 | 0.4 |
| WP_001229090.1 | ABC transporter protein CntA |  | P96 | 0.8 | 0.5 | 1.3 | 2.2 | 0.4 | 2.85 | ED6 | 0.9 | 1.8 | 9.3 | 0.6 | 0.1 |
| WP_000219068.1 | Catabolite control protein A CcpA | 0.3 | 3.9 | 0.6 | 0.6 | 1.7 | 1.6 | 0.6 | 1.73 | 0.8 | 0.6 | 1.1 | 0.6 | 0.9 | 1.7 |
| WP_000737654.1 | Manganese transport protein C (MntC) | 0.6 | 1.7 | 1.3 | 0.6 | 0.8 | 1.6 | 0.1 | 7.63 | 0.3 | 0.4 | 0.2 | 0.9 | 4.6 | 1.1 |
